# Staged reconstructive treatment for extensive irregular cicatricial alopecia after burn

**DOI:** 10.1097/MD.0000000000013522

**Published:** 2018-12-28

**Authors:** Songjia Tang, Xiaoxin Wu, Zhongxin Sun, Hanxiao Cheng, Haiyan Shen, Liang Tang, Jinghe Zhou, Ming Jia, Jinsheng Li, Jufang Zhang

**Affiliations:** a Department of Plastic Surgery, Affiliated Hangzhou First People's Hospital; b State Key Laboratory for Diagnosis and Treatment of Infectious Diseases, The First Affiliated Hospital, Zhejiang University School of Medicine, Hangzhou, China.

**Keywords:** cicatricial alopecia, hair transplantation, staged treatment, tissue expansion

## Abstract

For extensive irregular cicatricial alopecia after burn, effective and pleasing restoration of hair-bearing scalp remains challenging. In this article, the authors presented staged reconstructive treatment for extensive irregular cicatricial alopecia with the goal to achieve better and reliable results. A retrospective review of staged reconstructive treatment performed in 16 patients with extensive irregular cicatricial alopecia after burn was conducted. In stage 1, final flaps were designed at 1st. Tissue expanders were placed into the subgaleal plane and serially inflated with normal saline. In stage 2, scarring tissues were excised and the expanded hair-bearing flaps were advanced to the defect. Hair grafts were harvested from excessive hair-bearing scalps excised from the flaps and replanted. For patients with less satisfactory results, stage 3 was performed by hair transplantation. Cicatricial area, follicular unit density, survival rate of hair grafts, and patients’ satisfaction were measured before and after each stage. Thirteen patients received 3-stage treatment, and 3 received 2-stage treatment. Significant improvements in aesthetics and patient satisfaction were achieved in all the patients. No flap necrosis, implant exposure or hematoma was observed. Ideal, aesthetic, and reliable results could be obtained using staged reconstructive treatment for patients with extensive irregular cicatricial alopecia after burn.

## 1 Introduction

Cicatricial alopecia after burn usually appeared with extensive area, irregular borders, dispersed location, and precarious blood supply, which may be difficult to conceal and severely limit the patient's social rehabilitation.^[1]^ To achieve an ideal result, there are 2 basic principles. First, replacing like with like. Second, hair growth patterns, hair direction, and shape of hairlines should be naturally restored.^[2–4]^ Management strategies described in the literature include scalp reduction, hair transplantation, tissue extension, tissue expansion, and so on.^[5]^

Hair transplantation has been successfully demonstrated in extensive burn alopecia when the alopecia area is in relatively good condition. It is particularly indicated when hairline was involved.^[6,7]^ Tissue expansion is most commonly used for large area of cicatricial alopecia.^[1]^ Huge alopecia regions up to 50% of the scalp can be reconstructed using this method without an appreciable change in hair density.^[8]^ Scalp reduction and scalp extension were also applied when the area of burn alopecia is <100 cm^2^.^[1]^ In this article, we present staged reconstructive treatment for extensive irregular cicatricial alopecia after burn in an effort to achieving good functional and aesthetic outcomes.

## 2 Methods

### 2.1 Patients

A retrospective chart review was performed in this study. The patients gave informed consent, and the study was conducted in accordance with the ethical guidelines of the 1975 Declaration of Helsinki. Patients with extensive cicatricial alopecia after burn (scar size >120.0 cm^2^), who underwent staged reconstructive treatment from July 2006 to September of 2018, were included in this study. Patients with unstable cicatricial alopecia, scalp infection, and atrophic scar were excluded. There were 16 patients (10 women and 6 men) with an age range of 11 to 37 years (mean, 20.2 years) (Table 1).

**Table 1:**
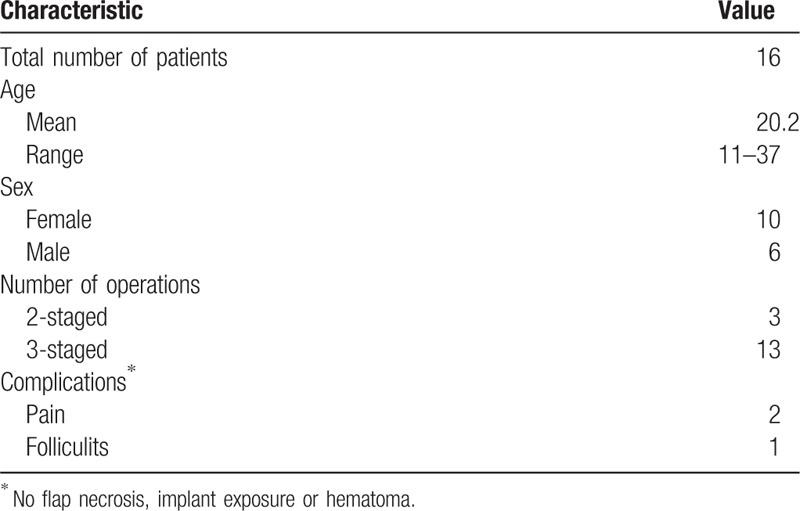
Patient demographics and complications.

### 2.2 Operative technique

**Figure 1:**
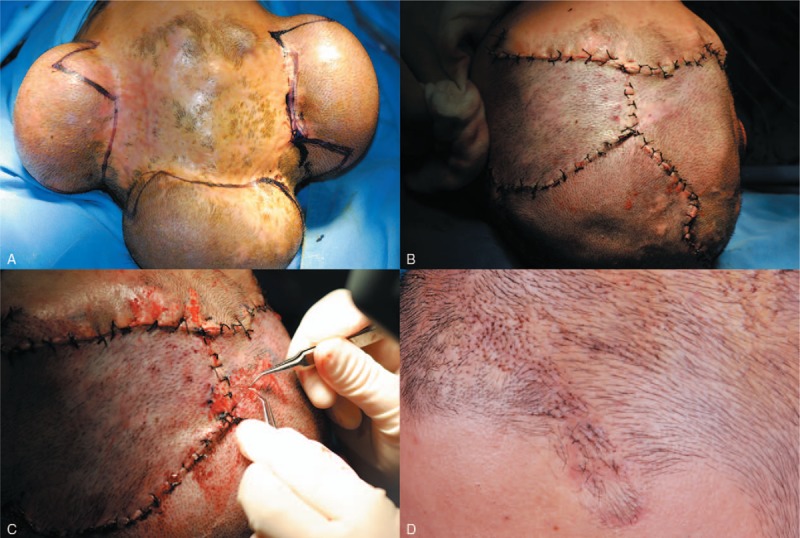
Staged reconstructive treatment. Stage 1: 3 expanders were serially inflated until adequate expansion (at least twice the surface area of alopecia) was attained (A). Stage 2: scarring tissues were excised and the expanded hair-bearing flaps were advanced to the defect (B). Hair grafts were harvested from excessive hair-bearing scalps excised from the flaps and replanted (C). For patients with less satisfactory results, hair transplantation was performed in stage 3 into hairline area, secondary scar and uncovered small alopecia areas (D).

Stage 1: Before the insertion of tissue expanders, final scalp were designed according to the size and location of alopecia. The design should combine principles of flap advancement based on at least 1 nominated vascular system used as the pedicle and the direction of hair growth match that of the major alopecia area. Then, 3 tissue expanders (Shanghai Winner Plastic Surgery Products, Shanghai, China, rectangular or reniform, 150–250 mL) were inserted into the subgaleal plane. Hairline area should be avoided. Expanders were filled up to 10% to 20% of their volume during the operation. The expanders were 1st inflated with normal saline 7 days after the wound had healed and proceeded with 3- to 5-day intervals until adequate expansion (at least twice the surface area of alopecia) was attained (Fig. 1A).

Stage 2: Four weeks after the last inflation, the expanders were removed. Before excision of the scarring tissue, expanded flaps were pulled toward the defect to estimate how much of the tissue can be removed without tension. Expanded hair-bearing flaps were advanced to the defect (Fig. 1B). Flaps were trimmed before advancement. Hair grafts were harvested in hair-bearing strips excised from the flaps and transplanted into hairline area and small dispersed uncovered alopecia area (Fig. 1C). Closed-suction drains were placed under the flap at the end of the operation for 48 hours to evacuate fluid and keep the flap adherent.

Stage 3: For patients with less satisfactory result, follicular unit extraction from occipital area was performed to restore residual alopecia area and secondary scar of previous operations (Fig. 1D). Cicatricial area and patients’ satisfaction were evaluated before and after each stage, survival rate of graft, follicular unit density in the reconstructed area was measured before and after the treatment. Patients were followed up at 3-month intervals for 1 year. After 1 year, patients were followed up annually.

## 3 Results

Thirteen patients underwent 3-stage treatment, 3 were satisfied with the result after stage 2 and did not receive procedure of stage 3 (Table 1). All patients were followed up for a mean duration of 26 months (range 10–35 months). No flap necrosis, implant exposure or hematoma occurred during the treatment. Two patients experienced mild pain and relieved with oral analgesics. One patients experienced donor-site folliculitis and were treated with oral antibiotics (Table 1). No further complications were noted in these cases. Cicatricial alopecia area decreased significantly after staged reconstructive treatment. Patients achieved an average reconstruction rate of 82.19% after stage 2 and an average reconstruction rate of 88.41% after stage 3 (Table 2). All patients achieved very satisfactory aesthetic results in terms of hair growth pattern, hair direction, and angle during follow-up periods. The following typical cases were reported.

**Table 2:**
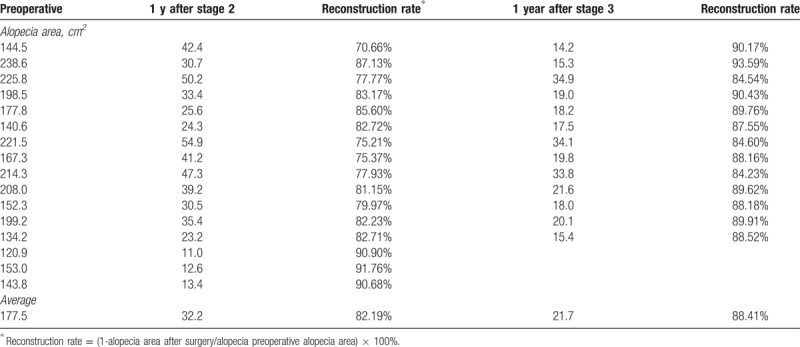
Evaluation of 16 patients after staged reconstructive treatment for extensive cicatricial alopecia after burn.

### 3.1 Case 1

**Figure 2:**
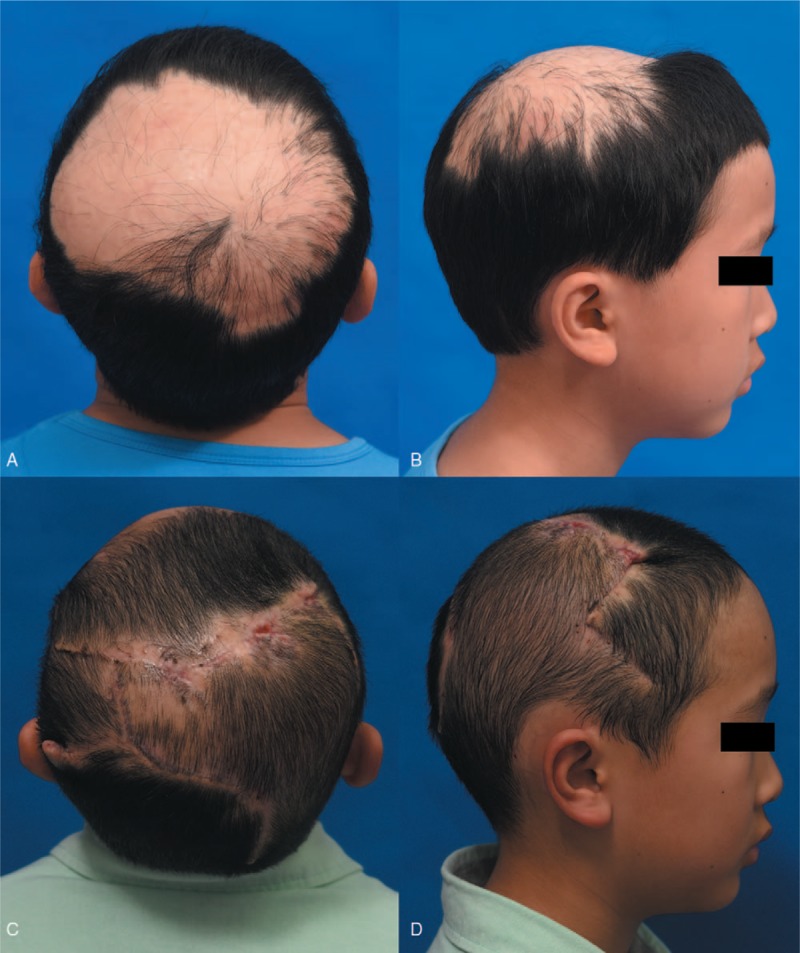
Case 1: A 11-year-old boy presented with cicatricial alopecia in the vertex after burn. Preoperative views (A, B). Six months after stage 2 surgery views (C, D).

A 11-year-old boy presented with cicatricial alopecia in the vertex after cooking oil burn with scar about 153.0 cm^2^ in size (Fig. 2A and B). Tissue expansion was performed in stage 1 with one 250 mL rectangular expander in right postauricular area, one 150 mL rectangular expander in the left postauricular area, and one 150 mL reniform expander in the frontal area. Flap advancement and hair transplantation were applied in stage 2. Six months after stage 2, 91.76% of the alopecia area was restored by hair-bearing scalp (Fig. 2C and D).

### 3.2 Case 2

**Figure 3:**
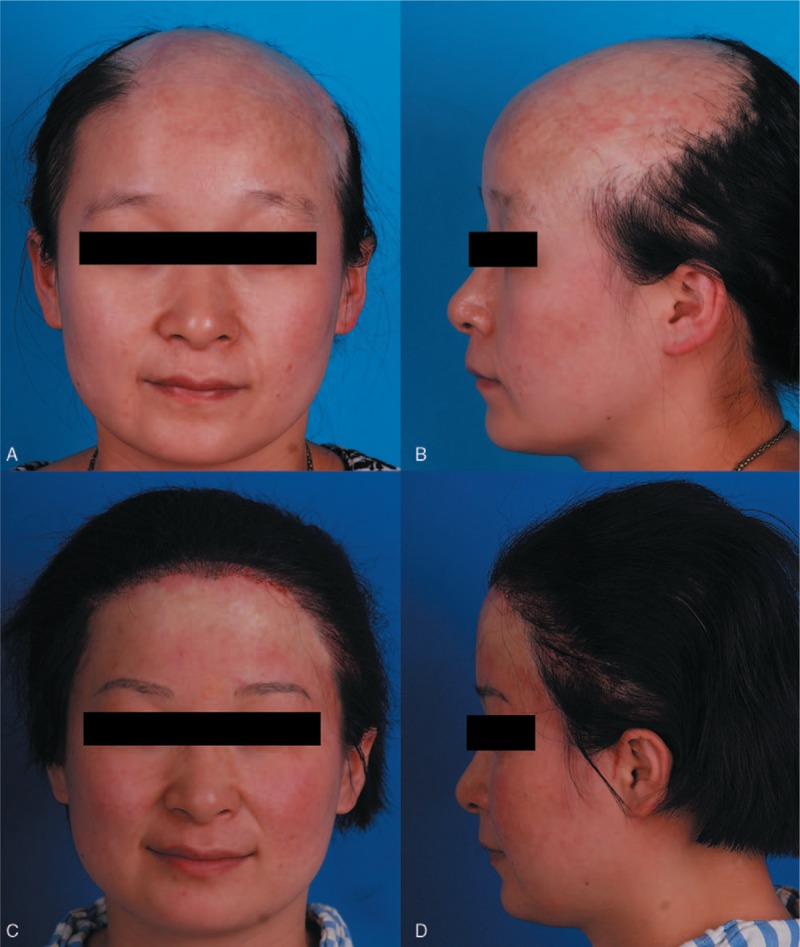
Case 2: A 26-year-old woman presented with cicatricial alopecia in the frontal area after burn. Preoperative views (A, B). Nine months after hairline restoration in stage 3 (C, D).

A 26-year-old woman presented with cicatricial alopecia in the frontal area after hot water burn with scar about 199.2 cm^2^ in size (Fig. 3A and B). In stage 1, one 250 mL rectangular expander in the vertex, one 200 mL reniform expander in the left occipital area, and one 200 mL rectangular expander in the right occipital area were inserted and inflated. Flap advancement and hair transplantation was performed in stage 2. Hairline restoration was performed in stage 3. Nine months after stage 3, 89.91% of the alopecia area and a natural hairline were restored (Fig. 3C and D).

## 4 Discussion

Cicatricial alopecia after burn can cause a large area of disfiguring deformities, which is usually difficult to conceal and requires surgical treatment.^[1]^ In most burn patients, alopecia may extend to different directions on the scalp, involve multiple parts, or disperse all over the scalp. Moreover, hair-bearing donor area is usually limited and the blood supply may be precarious in the scarring area, which makes it even more difficult to restore. Hair transplantation, scalp excision, tissue extension, or tissue expansion has been recommended for alopecia reconstruction according to the area, scar quality, and location of alopecia.^[5,9,10]^ Among which, hair transplantation and tissue expansion has been successfully demonstrated in large areas of burn alopecia.^[4,9,11]^

The field of hair transplantation is evolving rapidly and provides an effective means of scalp restoration.^[11,12]^ With attention to the hair direction, hair angle, and hair growth pattern during the procedure, hair transplantation could achieve a natural outlook with minimal complications. However, hair transplantation is usually time and labor consuming in order to achieve a dense hair pattern.^[13]^ The survival rate of follicular units (FUs) and final appearance may be compromised due to poor blood supply of alopecia area or operation by less experienced surgeons. There are also risks of permanent hair loss or less hair density due to necrosis or infection caused by inadequate perfusion.^[11]^

Tissue expansion has been an effective strategy to reconstruct large area of alopecia in a homogeneous pattern with a relatively low complication rate.^[14]^ It could provide large area of hair-bearing scalp with reasonable hair density, while the hair growth pattern, hair direction, hair angle, hairline, and sideburn shape were usually overlooked, which may lead to unexpected cosmetic results and leave trouble to secondary restoration.^[5,15]^ In fact, to achieve satisfactory results, repetition of expansion was usually necessary.^[16]^ The patient has to go through a prolonged course of treatment and accompany with “bubble head-like” appearance even longer.^[1]^

In patients with medium area of alopecia (<100 cm^2^), scalp reduction and scalp extension could also be considered.^[1]^ Scalp reduction is easy to practice and could achieve effective result. However, serial excision may result in a vertical scar which was difficult to hide. Scalp extension could eliminate the stretch-back phenomenon postoperatively and reduce the number of repetitions.^[17]^ Nevertheless, complication rate of this procedure is higher as the constant use of foreign material.

Successful restoration for extensive cicatricial alopecia after burn requires accurate evaluation of the alopecia, combination of procedures, and staged treatments. Preoperative evaluation of the quality of scar, the size of defect, the patient's healing characteristics, vascular supply, availability of donor hair, and the orientation of hair growth are important when attempting to reconstruct the affected area. In most of our patients, the scar is of poor quality. An average reconstruction rate of 82.19% could be achieved after stage 2 and an average reconstruction rate of 88.41% after stage 3. In our experience, the duration of burn scar also would not affect the outcome of this treatment as long as the cicatricial alopecia is stable.

In stage 1 and stage 2, multiple expanders were used to gain the greatest amount of expansion. The expanded flaps should be designed and advanced to recreate normal hair patterns and directions. Restoration of natural hair pattern is the primary consideration rather than complete covering of cicatricial alopecia. Before excision of the scarring tissue, expanded flaps should be pulled toward the defect to estimate how much of the tissue can be removed without tension. It should be noticed not to overexcise the excessive parts of expanded tissue as small dog ears would disappear in the recovery.^[4]^ Flaps should be sutured in a relaxed tonus to minimize secondary scar and prevent hair loss. Scar excision and hair transplantation in small scale could also be performed in the same stage. Hair transplantation should be performed according to the perfusion of the recipient area. “Trichophytic” approach should be utilized in the final closure.^[18]^

Stage 3 was necessary for most patients. Follicular unit extraction was performed to restore residual alopecia area and secondary scar of previous operations, which shortened the period of treatment and phase of disfigured appearance compared with repetitive expansion. Density of at most 20 to 25 FU/cm^2^ should be performed to prevent necrosis or infection caused by inadequate perfusion.^[5]^ In hairline area, 1-hair follicular units should be used to ensure a natural appearance, while in other areas, 2- and 3-haired follicular units should be utilized to create higher hair concentration.

## 5 Conclusion

For the purpose of achieving ideal and reliable aesthetic results for extensive irregular cicatricial alopecia, a newly designed staged reconstructive treatment was performed. Although the number of cases was limited, we believe that good functional and effective cosmetic results could be obtained by this approach.

## Acknowledgment

The authors thank Shufen Du for contributing to creation of the figures for this project.

## Author contributions

**Conceptualization:** Hanxiao Cheng.

**Data curation:** Songjia Tang, Xiaoxin Wu, Zhongxin Sun, Hanxiao Cheng, Haiyan Shen, Ming Jia, Jinsheng Li, Jufang Zhang.

**Formal analysis:** Jufang Zhang.

**Funding acquisition:** Jinghe Zhou, Jufang Zhang.

**Investigation:** Songjia Tang, Hanxiao Cheng, Haiyan Shen, Liang Tang, Jinghe Zhou, Ming Jia, Jinsheng Li, Jufang Zhang.

**Methodology:** Songjia Tang, Hanxiao Cheng, Haiyan Shen, Liang Tang, Jinghe Zhou, Jinsheng Li, Jufang Zhang.

**Project administration:** Jufang Zhang.

**Resources:** Songjia Tang, Hanxiao Cheng, Haiyan Shen, Liang Tang, Jinghe Zhou, Jinsheng Li, Jufang Zhang.

**Supervision:** Jinsheng Li, Jufang Zhang.

**Validation:** Xiaoxin Wu, Zhongxin Sun, Hanxiao Cheng, Haiyan Shen, Jinsheng Li, Jufang Zhang.

**Writing – original draft:** Songjia Tang, Xiaoxin Wu, Zhongxin Sun, Hanxiao Cheng, Haiyan Shen, Liang Tang, Jinghe Zhou, Ming Jia, Jinsheng Li, Jufang Zhang.

**Writing – review & editing:** Songjia Tang, Xiaoxin Wu, Zhongxin Sun, Jufang Zhang.
